# Synchronous neuroendocine liver metastases in comparison to primary pancreatic neuroendocrine tumors on MRI and SSR-PET/CT

**DOI:** 10.3389/fonc.2024.1352538

**Published:** 2024-05-31

**Authors:** Annie Horng, Maria Ingenerf, Frank Berger, Denise Steffinger, Johannes Rübenthaler, Matthias Zacherl, Vera Wenter, Jens Ricke, Christine Schmid-Tannwald

**Affiliations:** ^1^ Department of Radiology, University Hospital, Ludwig-Maximilians-Universität (LMU) Munich, Munich, Germany; ^2^ Department of Nuclear Medicine, University Hospital, Ludwig-Maximilians-Universität (LMU) Munich, Munich, Germany; ^3^ European Neuroendocrine Tumor Society (ENETS) Centre of Excellence, Interdisciplinary Center of Neuroendocrine Tumours of the GastroEnteroPancreatic System at the University Hospital of Munich (GEPNET-KUM), University Hospital, Ludwig-Maximilians-Universität (LMU) Munich, Munich, Germany

**Keywords:** prognosis, multiparametric magnetic resonance imaging (mpMRI), positron - emission tomography, pancreatic neuroendocrine tumor, synchronous liver metastases

## Abstract

**Background:**

The study aimed to compare and correlate morphological and functional parameters in pancreatic neuroendocrine tumors (pNET) and their synchronous liver metastases (NELM), while also assessing prognostic imaging parameters.

**Methods:**

Patients with G1/G2 pNET and synchronous NELM underwent pretherapeutic abdominal MRI with DWI and 68Ga-DOTATATE/TOC PET/CT were included. ADC (mean, min), SNR_art and SNT_T2 (SNR on arterial phase and on T2) and SUV (max, mean) for three target NELM and pNET, as well as tumor-free liver and spleen (only in PET/CT) were measured. Morphological parameters including size, location, arterial enhancement, cystic components, T2-hyperintensity, ductal dilatation, pancreatic atrophy, and vessel involvement were noted. Response evaluation used progression-free survival (PFS) with responders (R;PFS>24 months) and non-responders (NR;PFS ≤ 24 months).

**Results:**

33 patients with 33 pNETs and 95 target NELM were included. There were no significant differences in ADC and SUV values between NELM and pNET. 70% of NELM were categorized as hyperenhancing lesions, whereas the pNETs exhibited significantly lower rate (51%) of hyperenhancement (p<0.01) and significant lower SNR_art. NELM were qualitatively and quantitatively (SNR_T2) significantly more hyperintense on T2 compared to pNET (p=0.01 and p<0.001). NELM of R displayed significantly lower ADCmean value in comparison to the ADC mean value of pNET (0.898 versus 1.037x10^-3^mm²/s,p=0.036). In NR, T2-hyperintensity was notably higher in NELM compared to pNET (p=0.017). The hepatic tumor burden was significantly lower in the R compared to the NR (10% versus 30%).

**Conclusions:**

Arterial hyperenhancement and T2-hyperintensity differ between synchronous NELM and pNET. These findings emphasize the importance of a multifaceted approach to imaging and treatment planning in patients with these tumors as well as in predicting treatment responses.

## Introduction

Neuroendocrine tumors originating in the pancreas (pNET) are exceedingly rare, occurring at a rate of 0.48 cases per 100,000 individuals, and they make up just 1% of all pancreatic tumors (1–3). Because they grow slowly and often present with vague symptoms, particularly in cases of non-functional pNETs, they are frequently diagnosed at an advanced stage, with neuroendocrine liver metastases (NELM) detected in approximately 64% to 70% of cases (4). This limits the feasibility of curative surgical removal, leading therapy strategies to focus primarily on symptom management and restraining tumor progression (5).

Advanced imaging methods, such as magnetic resonance imaging (MRI) and positron emission tomography/computed tomography (PET/CT) utilizing somatostatin receptor (SSR) targeting, have become indispensable for diagnosing and tracking neuroendocrine tumors. Morphological aspects in imaging, such as arterial enhancement, the presence of cystic/necrotic areas, and T2-weighted hyperintensity, along with functional features such as apparent diffusion coefficient (ADC) values and standardized uptake values, provide crucial insights into tumor characteristics, blood supply, and tissue composition.

Given that treatment choices for pNETs with synchronous liver metastases depend on factors like tumor grade, the extent of liver involvement, vascularization, SSR uptake, symptoms, and the patient’s overall health, it’s imperative to incorporate both morphological and functional imaging data with clinical and pathological assessments.

Studies have investigated imaging characteristics of primary pNETs on pre-therapy imaging in patients to appraise tumor grading (6, 7) or identify prognostic factors for outcomes (8–10). Several retrospective investigations have demonstrated the utility of DWI and ADC measurements in assessing tumor response in liver metastases/liver tumors and even predicting response based on initial ADC values (11–14). Studies have also illustrated the prognostic and predictive significance of imaging parameters derived from SSR-PET/CT in patients undergoing peptide receptor radionuclide therapy (PRRT) or octreotide treatment for NETs (15–18). However, regarding response assessment Ingenerf et al. demonstrated that there are differences between pNET and its metastases: ADCmin values of NELM decreased significantly in responders following the initiation of everolimus treatment, whereas ADC values in nonresponders increased. Conversely, ADC (both minimum and mean) of pNETs tended to increase in responders, while there was no change observed in nonresponders, indicating distinct behavior between pNETs and their NELM counterparts (19).

Currently, to our knowledge, no existing research has examined morphological and functional imaging parameters in patients with pNETs and synchronous NELM to determine whether disparities exist in baseline imaging and whether there are prognostic indicators when curative treatment is no longer feasible.

The primary goal of this study is to compare and correlate morphological and functional parameters between pNETs and their synchronous NELM and assess imaging-based prognostic factors.

## Material and methods

In this retrospective study, we examined consecutive patients with newly diagnosed G1/G2 pancreatic neuroendocrine tumors who had synchronous liver metastases between January 2009 and November 2020. These patients had undergone pretherapeutic imaging, including MRI with DWI and either 68Ga-DOTA-TATE or 68Ga-DOTA-TOC PET/CT scans at our facility. At the time of imaging, the patients were not receiving any treatment, neither drug nor surgical. Patients with G3 pNET were excluded because they routinely underwent FGD PET/CT in addition to liver MRI.

**Figure 1:**
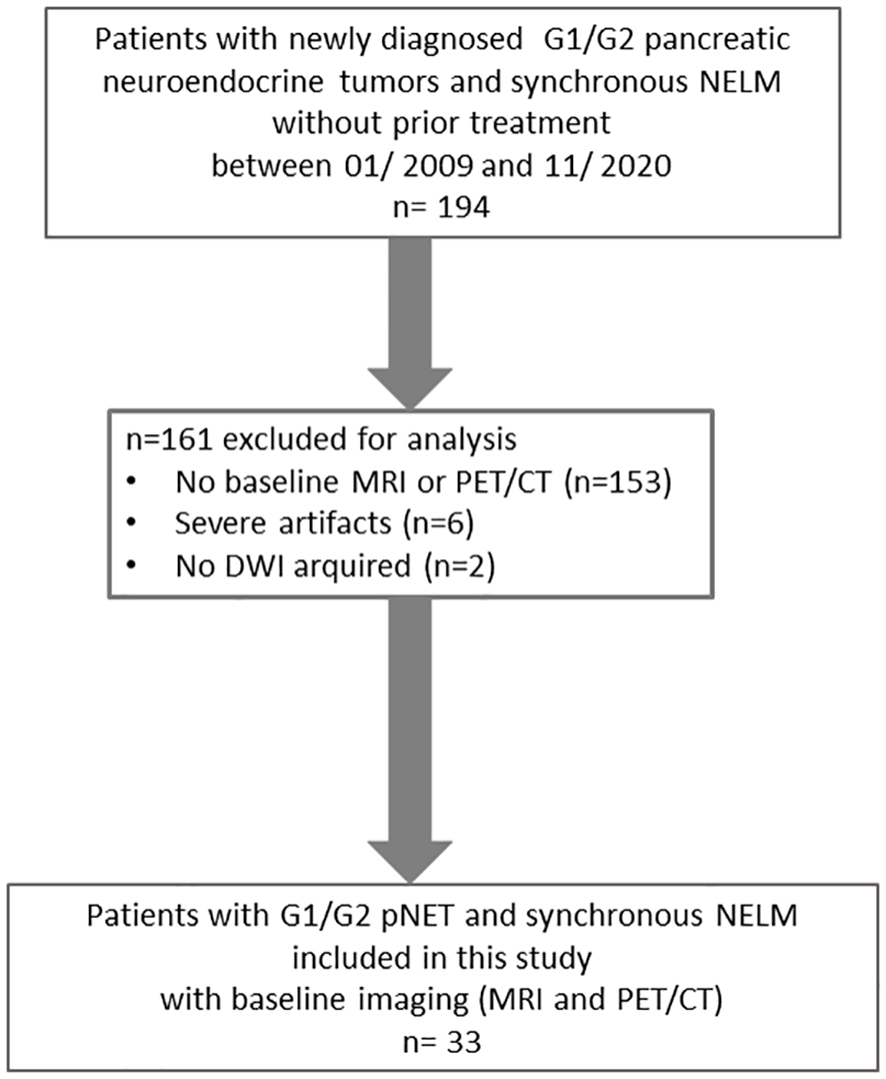
Flow-chart.

The inclusion criteria necessitate a minimum size of 1 cm for both the primary pancreatic tumor and its metastases, with the exclusion of cases impacted by notable motion artifacts (**Figure 1**).

Approval for this study was obtained from the local research ethics committee at LMU University Munich, and the need for written informed patient consent was waived due to the retrospective design.

### MR imaging

Magnetic resonance (MR) examinations were conducted using a 1.5 Tesla MR system, specifically the Magnetom Avanto and Magnetom Aera by Siemens Healthcare in Erlangen, Germany, as well as the Ingenia S by Philips Healthcare in Hamburg, Germany. These MR machines employed a phased-array coil for signal reception.

The standard imaging protocol encompassed the following sequences: (1) an unenhanced T1-weighted gradient-echo (GRE) sequences, both in-phase and out-of-phase, (2) a single-shot T2-weighted sequence, (3) a T1-weighted 3D GRE sequence with fat suppression, performed before contrast injection and at 20, 50, and 120 seconds after intravenous administration of contrast agent (Gd-EOB-DTPA; Primovist or Eovist; Bayer Schering Pharma, Germany) at a dosage of 25 µmol/kg body weight, (5) a multishot T2-weighted turbo spin echo sequence with fat saturation, (6) diffusion-weighted sequences with b-values of 50 and 800 s/mm², (7) following a 15-minute delay, an additional T1-weighted GRE sequence with fat saturation, as well as (8) a fat-suppressed T1-weighted VIBE 3D GRE sequence identical to those previously performed. Parallel imaging with an acceleration factor of 2 was applied to all sequences. Apparent diffusion coefficient (ADC) maps were generated, encompassing data from all b-values.

### PET/CT

Whole-body PET/CT scans were conducted in three-dimensional mode, with a duration of 3 minutes per bed position. These scans were performed using either a Biograph 20, 64 or TruePoint PET/CT scanner from Siemens Healthcare in Erlangen, Germany.

The imaging process commenced 60 minutes after the intravenous administration of approximately 200 MBq of 68Ga-DOTA-TATE, along with, if feasible, 20mg of furosemide. PET/CT scans involved obtaining diagnostic CT images of the neck, thorax, abdomen, and pelvis. The CT parameters were set at 100–190 mAs, 120 kV, with a collimation of 2 x 5 mm and a pitch of 1.5. Additionally, an iodine-based contrast agent (Ultravist 300TM; provided by Bayer Healthcare in Berlin, Germany) was intravenously injected at a rate of 2.5 mL/s, at a dose of 1.5 mL/kg body weight, with a 50-second delay to visualize the portal venous phase of the liver. CT scans also served for PET attenuation correction. Standardized uptake values (SUV) were calculated using the patient’s body weight (SUVbw).

### Image analysis

MRI images and PET/CT were reviewed by two radiologists in consensus, and in two separate sessions. None of the readers were aware of patients’ follow-up data. Three target liver lesions and pancreatic NET were defined in consensus for each patient. Morphological MR imaging parameters were assessed for all target lesions, which included up to three liver metastases and pancreatic neuroendocrine tumors (pNETs). These parameters encompassed: 1. Location, 2. Size, 3. Percentage of arterial enhancement (visually assessed), 4. Hyperintensity on T2-weighted images, categorized as 0 (none), 1 (low), 2 (intermediate), or 3 (strong), 5. Presence of cystic or necrotic areas within the lesions.

Arterial hypervascularization was defined as arterial enhancement of the lesion exceeding 50%. Furthermore, for pNETs, the following factors were documented:

- Ductal dilatation in the pancreas, reported when the main pancreatic duct exceeded 4 mm in diameter.
- Pancreatic parenchyma atrophy, defined by an observed reduction in pancreatic volume beyond the expected.
- Vessel involvement.

The size of the lesions was measured as the longest diameter (LD) during the hepatocyte-specific contrast phase on the slice displaying the largest tumor extent. This measurement was averaged across the three target lesions for each patient.

To calculate the apparent diffusion coefficient (ADC), circular regions-of-interest (ROIs) were delineated on the slice with the largest extent of the target lesions in diffusion-weighted imaging (DWI) images. Care was taken to exclude structures near the lesion’s edge to prevent partial volume effects. These ROIs were then applied to the corresponding slice of the ADC map to compute intralesional ADC values, which included ADCmin and ADCmean, expressed as 10^-3^ mm^2^/s.

On non-enhanced T2w and T1w + contrast enhanced images (arterial phase), SI values were recorded by drawing ROIs of all selected metasases and pNET as large as possible. In addition, image node was measured as the standard deviation of a ROI in the left corner outside the body volume. SNR was calculated as SNR= SI_lesions/Noise (standard deviation of a region-of-interest outside).

68Ga-DOTA-TATE uptake was measured as maximum and mean SUV by semi-quantitatively positioning a circular VOI in the predefined target lesion. Also, SUVmax and SUVmean of non-tumorous liver and spleen parenchyma were assessed to calculate tumor to organ ratios with tumor-to-spleen (T/S) ratio and tumor-to-liver (T/L) ratio (including SUVmax/SUVmax, SUVmax/SUVmean and SUVmean/SUVmean).

### Standard of reference and response to treatment

Diagnosis of pNET was confirmed by histopathology, and Ki-67 labelling index of the primary tumor was obtained for all included patients. Tumor grading was rated according to 2017 WHO Tumor Classification Guideline. In addition, the therapy following the pretherapeutic imaging until progression or for the next 24 months was documented for each patient by a 3rd radiologist.

Progression-free survival (PFS) was used as a parameter for treatment response, calculated in months from the time of initiation of therapy until progression, as evaluated by the local interdisciplinary tumor board through the assessment of all performed imaging studies (CT, PET/CT, MRI). Responder were defined as patients with PFS > 24 months and non-responders (NR) as patients with PFS ≤ 24 months respectively.

### Statistical analysis

Statistical analysis was conducted using commercially available software, specifically Graphpad Prism Version 6 in San Diego, California, and SPSS Version 25 in Chicago, Illinois. The threshold for statistical significance was set at p ≤ 0.05.

We visually examined the frequency distribution through histograms. We determined whether continuous variables followed a normal distribution by visually examining their frequency distribution through histograms. We presented the data as either mean values with standard deviation (SD) or median values with interquartile range (IQR).

For comparing means of two continuous variables that followed a normal distribution, Student’s t-test was utilized. In cases where the distribution was not normal, the Mann-Whitney U test was performed to assess differences between groups.

Additionally, a correlation analysis was conducted between the morphological parameters and the quantitative parameters. A correlation coefficient above 0.5 was defined as a higher degree of correlation. Area under the curve (AUC) was calculated from receiver operating characteristics (ROC) analysis and used to define optimal thresholds and to compare pNET and NELM different parameters (SNR on T2 and arterial phase).

## Results

### Patients

In this retrospective study, a total of 33 patients were included, comprising 14 females and 19 males, with an average age of 65 years. Among these patients, there were 33 pNETs and a combined total of 95 target NELM.

However, in three patients, we were unable to evaluate DWI due to motion artifacts, and in three other patients, PET/CT assessment was not possible due to artifacts. The baseline MRI was obtained within an average of 8 ± 31 days before or after the PET/CT scan.

The majority of the patients with neuroendocrine tumors (NET) had G2 tumors (n=30), while a smaller number had low-grade tumors (G1) with a count of three. Notably, no high-grade (G3) tumors were included in this study. Detailed patient characteristics are listed in **Table 1**.

**Table 1:** Patient characteristics.

Sex	
***Male***	19 (58%)
***Female***	14 (42%)
**Median age, years (range)**	65 (42–92)
Grading	
***G1***	3 (5%)
***G2***	30 (77%)
**median Ki-67 (ng/ml) (range)**	10 (1 – 20)
pNET Location	
**head**	13 (39%)
**corpus**	7 (22%)
**tail**	13 (39%)
Multimodal therapy until tumor progression or within the next 24 months	
***pNET resection***	9 (27%)
***Somatostatin analogs***	17 (52%)
***chemotherapy***	12 (36%)
***immunotherapy***	1 (3%)
***Targeted therapy***	15 (45%)
***PRRT***	6 (18%)
***liver targeted therapy***	3 (9%)

### Pretherapeutical measurements, differences and correlations between NELM and pNET

**Figure 2:**
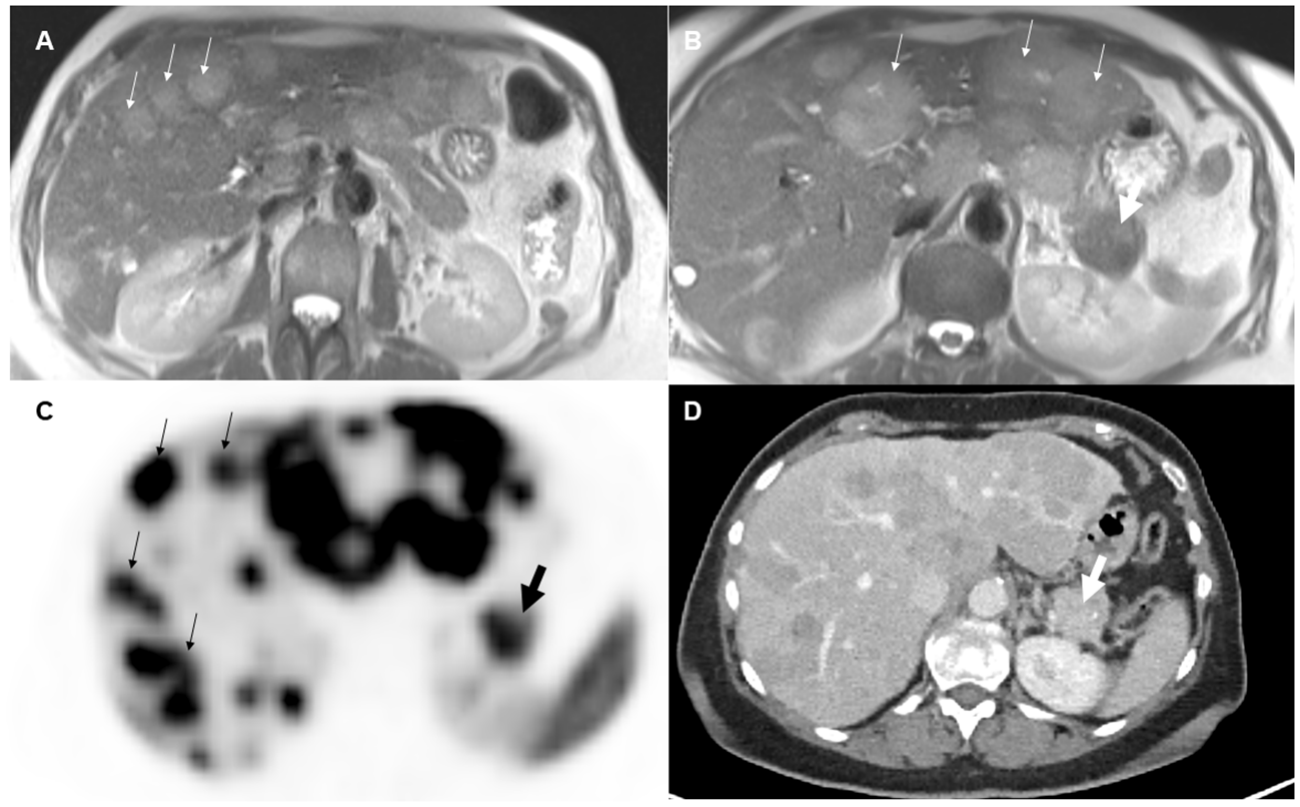
70-year-old female with pancreatic neuroendocrine tumor and synchronous neuroendocrine liver metastases. On T2-weighted images **(A, B)**, the liver metastases (small arrows) appeared significantly more hyperintense than the primary tumor (thick arrow) in the pancreatic tail. On pretherapeutic positron emission tomography-computed tomography examination **(C, D)**, 68Ga-DOTA-TATE tracer uptake was similar between the metastases and the pancreatic neuroendocrine tumor (SUVmean NELM: 35, SUVmean pNET: 32).

The pretherapeutic imaging parameters, as summarized in **Table 2**, revealed that there were no noteworthy alterations in ADC values and SUV values between the NELM and pNET. NELM were significantly more hyperintense in the T2-weighted sequence compared to pNET (p=0.01) (**Figure 2**). Correspondingly, SNR_T2 of NELM were significantly higher than SNR_T2 of pNET (p< 0.001).

**Table 2:** Pretherapeutic imaging parameters and comparison between NELM and pNET.

	Pretherapeutic [mean ( ± SD)]	p-Value (comparison between NELM and pNET)
NELM Size (mm)^1^	28 ( **±** 10)	**<0.01**
NELM hyperenhancement (%) NELM percentual arterial enhancement **^1^** NELM cystic/necrotic components**^1^** NELM hyperintensity (%)**^1^** NELM T2 hyperintensity (grading)**^1^** NELM ADCmean**^1^** NELM ADCmin**^1^** NELM SUVmax**^1^** NELM SUVmean**^1^** NELM SNR_T2**^1^** NELM SNR_art**^1^** pNET Size (mm) pNET hyperenhancement (%) pNET percentual arterial enhancement (%) pNET cystic/necrotic components pNET T2 hyperintensity (%) pNET T2 hyperintensity (grading)	70% 62 ( **±** 33) 0.3 ( **±** 0.4) 55% 1.7 ( **±** 0,9) 0.937 ( **±** 0.237) 0.479 ( **±** 0.132) 33 ( **±** 15) 25 ( **±** 11) 137 ( **±** 91) 83 ( **±** 57) 47 ( **±** 21) 51% 47 ( **±** 35) 0.3 ( **±** 0,5) 30% 1.2 ( **±** 0,9)	**<0.01** **0.01** 0.06 **0.01** 0.10 0.20 0.34 0.47 **<0.001** **<0.001**
pNET ADCmean pNET ADCmin	1.002 ( **±** 0.264) 0.517 ( **±** 0.255)	
pNET SUVmax	35 ( **±** 23)	
pNET SUVmean pNET SNR_T2 pNET SNR_art Liver ADC mean	25 ( **±** 15) 98 ( **±** 63) 64 ( **±** 44) 0.990 ( **±** 0.201)	
Liver SUVmax	7 ( **±** 2)	
Liver SUVmean	5 ( **±** 2)	
Spleen SUVmax	21 ( **±** 6)	
Spleen SUVmean	18 ( **±** 6)	
NELM**^1^**/L (max/max) NELM**^1^**/S (max/max) NELM**^1^**/L (max/mean) NELM**^1^**/S (max/mean) NELM**^1^**/L (mean/mean) NELM**^1^**/S (mean/mean) pNET/L (max/max)	5 ( **±** 2) 2 ( **±** 1) 7 ( **±** 4) 2 ( **±** 1) 5 ( **±** 3) 2 ( **±** 1) 5 ( **±** 3)	0.85 0.85 0.84 0.81 0.92 0.90
pNET/S (max/max)	2 ( **±** 1)	
pNET/L (max/mean)	7 ( **±** 5)	
pNET/S (max/mean)	2 ( **±** 1)	
pNET/L (mean/mean)	5 ( **±** 3)	
pNET/S (mean/mean) hepatic tumor burden	1 ( **±** 1) 15 ( **±** 25)	

**^1^** averaged for up to three target NELM per patient.

Values with statistical significance (p ≤ 0.05) are in bold.

**Figure 3:**
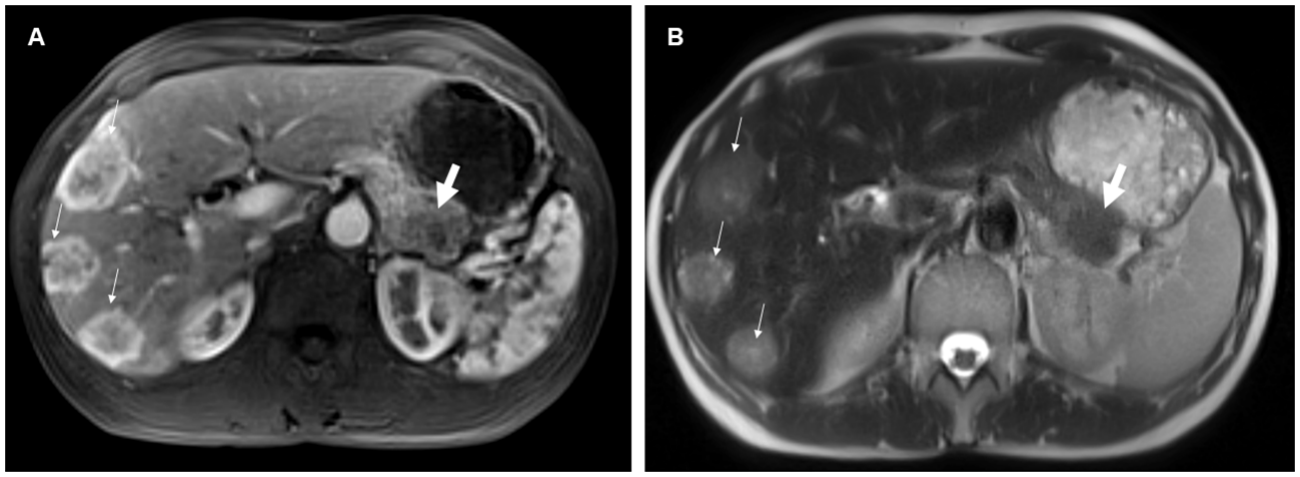
54-year-old female with pancreatic neuroendocrine tumor and synchronous neuroendocrine liver metastases. On contrast-enhanced T1-weighted images in the arterial phase **(A)**, the liver metastases (small arrows) predominantly exhibited hyperenhancement, whereas the pancreatic primary tumor (thick arrow) was hypoenhancing compared to the liver metastases. Additionally, the neuroendocrine liver metastases were more hyperintense on the T2-weighted image **(B)** compared to the primary tumor.

70% of NELM were rated as hyperenhancing lesions with a mean percentage of arterial vascularization of 62%, while the pNET were rated significantly less as hyperenhancing lesions (51%) with a mean percentage of arterial vascularization of 47% (p<0.01) (**Figure 3**). Correspondingly, SNR_art of NELM were significantly higher than SNR_art of pNET (p< 0.001). As expected, ROC analysis using SNR values failed to discriminate pNET and its NELM (AUC < 0.7 using SNR_T2 and SNR_art). The mean size of NEML was significantly smaller than the mean size of pNET (28mm vs. 47mm; p>0.001), however the metastases demonstrated similarly often cystic/necrotic components compared to pNET (p=0.01) (**Table 2**).

In 17/33 (52%) of patients a pancreatic duct dilatation was found and a vascular involvement of the pNET was found in 20/33 (61%) of the patients.

Moderate correlations were identified between the presence of cystic/necrotic changes of NELM and ADC mean of NELM (r=0.51). Otherwise, higher degree correlations were not observed between the morphological characteristics and the quantitative measurements of ADC values and SUV values in NELM.

In pNET, evidence of cystic changes and T2 hyperintensity showed higher-grade correlations with the mean ADC value of pNETs (r=0.51 and 0.69, respectively). Moreover, as expected, a robust correlation was observed between the SUVmax value within the primary pNET and the mean SUVmean value of the pNET (r=0.90).

Significant correlations were also identified between SUVmax of NELM and SUV max of pNET (r=0.53) and between SUVmean of NELM and SUVmean of pNET (r=0.52). Furthermore, there was notable correlation, particularly between the mean ADC value of metastases and pNET (r=0.51).

### Response prediction according to PFS > 24 months

Out of the total of 33 patients, 19 experienced a progression-free survival (PFS) longer than 24 months, categorizing them as responders, while the remaining 14 patients were classified as non-responders.

Comparing NELM versus pNET, responders exhibited a significantly smaller mean size of NELM when compared to the primary pNET [26mm (NELM) versus 50mm (pNET) p>0.001]. Conversely, there was no significant difference in size between NELM and pNET in non-responders [32mm (NELM) versus 44mm (pNET), p=0.051] Additionally, NELM of responders displayed significantly lower ADCmean value in comparison to the ADC mean value of pNET (0.898 versus 1.037 x 10^-3^ mm²/s, p=0.036).

In contrast, the ADC mean values of NELM and pNET were not significantly different in non-responders (0.981 versus 0.961 x 10^-3^ mm²/s, p=0.382).

In the case of non-responders, T2 hyperintensity was notably higher in NELM compared to pNET (p=0.017).

Arterial enhancement, location of the primary, ductal dilatation or pancreatic atrophy did not significantly differ between responders and non-responders.

The hepatic tumor burden was significantly lower in the responders compared to the non-responders (10% versus 30%). Comparing responders and non-responders regarding NELM only and pNET only there were no other significant distinctions. Further detailed assessments can be found in **Tables 3**, **4**.

**Table 3:** Pretherapeutic imaging parameters of responders and non-responders.

	Responder [mean ( ± SD)]	Non-responder [mean ( ± SD)]
NELM Size (mm)^1^	26 ( **±** 9)	32 ( ± 11)
NELM hyperenhancement (%) NELM percentual arterial enhancement **^1^** NELM cystic/necrotic components**^1^** NELM T2 hyperintensity (grading)^1^ NELM ADCmean**^1^** NELM ADCmin**^1^** NELM SUVmax**^1^** NELM SUVmean**^1^** pNET Size (mm) pNET hyperenhancement (%) pNET percentual arterial enhancement (%) pNET cystic/necrotic components pNET T2 hyperintensity (grading)	68% 64 ( **±** 33) 0.4 ( **±** 0.4) 1.6 ( **±** 0,9) 0.898 ( **±** 0.245) 0.497 ( **±** 0.176) 36 ( **±** 16) 26 ( **±** 11) 50 ( **±** 22) 53% 49 ( **±** 36,7) 0.3 ( **±** 0,5) 1.3 ( **±** 0,9)	71% 58 ( ± 32) 0.4 ( ± 0.4) 1.7( ± 0.9) 0.981 ( ± 0.296) 0.459 ( ± 0.181) 29 ( ± 12) 23 ( ± 11) 44 ( ± 19) 50% 44 ( ± 34) 0.3 ( ± 0.6) 1.2 ( ± 1.0)
pNET ADCmean pNET ADCmin	1.037 ( **±** 0.249) 0.520 ( **±** 0.276)	0.961 ( ± 0.275) 0.514 ( ± 0.229)
pNET SUVmax	40 ( **±** 27)	28 ( ± 14)
pNET SUVmean hepatic tumor burden (%)	28 ( **±** 16) 30%	20 ( ± 12) 10%

**Table 4:** Comparison between responders and non-responders.

	Size	Arterial vascul-arization	Cystic/ necrotic compo-nents	T2-hyper-intensity (grading)	ADC mean	ADC min	SUV max	SUV mean
**Nonresponder:** NELM versus pNET	0.051	0.053	0.246	**0.017**	0.382	0.220	0.369	0.319
**Responder:** NELM versus pNET	**< 0.001**	0.052	0.406	0.128	**0.036**	0.355	0.275	0.376
**NELM:** Responder versus non-responder	0.053	0.312	0.302	0.378	0.213	0.291	0.158	0.208
**pNET:** responder versus non-responder	0.202	0.360	0.437	0.390	0.225	0.478	0.325	0.344

Values with statistical significance (p ≤ 0.05) are in bold.

## Discussion

In this study we found no significant differences in ADC values and SUV values between NELM and pNET but notable correlations between SUV values of NELM and pNET, suggesting a similar SSR expression between the primary tumor and liver metastases. Also, a moderate correlation was found between mean ADC values of NELM and pNET. These findings collectively emphasize the importance of considering both metabolic activity (SUV values) and tissue diffusion properties (ADC values) when assessing and understanding the relationship between primary tumors and their liver metastases in patients with NELM and pNET. While we didn’t identify significant differences in these parameters, the correlations observed hint at the potential for shared characteristics that could impact disease progression and treatment response. Further research is needed to delve deeper into these associations and their clinical implications.

It is essential to recognize the disparities in arterial enhancement between NELM and pNET as they can have significant implications for treatment planning. Ronot and colleagues conducted an assessment of enhancement differences in NELM based on their primary origin and found that 70% of NELM were hypervascular (20). Notably, there was no significant difference in hypervascularity between pancreatic NLMs and enteric NLMs. In our study group, we also observed that 70% of metastases exhibited hypervascularization; in addition, we found that pNET exhibited significantly less hyperenhancement (51%) and showed a significant lower SNR_art. The hypervascularization of tumors, coupled with the frequent overexpression of growth factors and constitutive overactivation of the PI3K-Akt-mTOR signaling pathway in neuroendocrine carcinomas, provides a rationale for exploring new molecularly targeted therapeutic approaches (21–23). It is worth noting that the impact of targeted cytostatic agents differs from traditional cytotoxic treatments, as it does not result in immediate cell death or significant size reduction. When evaluating radiological responses with antiangiogenic effects, it is crucial to recognize that NELM exhibit distinct perfusion characteristics compared to primary pNET.

Furthermore, in our study, we observed that NELM also displayed higher T2 hyperintensity compared to pNET, indicating differences in tissue composition. These findings align with the widely accepted knowledge in the literature concerning NET liver metastases, where they have been described as having “fluid-like signal intensity” (24), resembling a “pseudo-angiomatous” or “hemangioma-like” appearance (24–26). In a study examining the MRI appearance of carcinoid tumors and their metastases in 12 patients with 146 liver metastases, the authors reported that 75% of all liver lesions appeared moderately hyperintense on T2-weighted imaging (27). In a study by Sommer et al., it was found that 39% of NELM displayed markedly hyperintense signals in T2-weighted imaging, similar to our findings (28). While our analysis, like that of Sommer et al., was patient-based rather than lesion-based, our series revealed a higher prevalence (55%) of markedly hyperintense signals in T2-weighted imaging among NELM (28). In our study, only 30% of pNET cases exhibited hyperintensity on T2-weighted imaging. It is worth noting that Manfredi et al. reported that 82.2% of neuroendocrine tumors appeared hyperintense on T2-weighted MRI. However, in their study, they did not make further distinctions based on signal intensity characteristics in T2-weighted imaging.

In addition, we found that NELM were also significantly hyperintense in the T2-weighted sequence compared to pNET. In a study conducted by Thüring et al., the T2 signal intensity of metastases from colorectal carcinoma and breast cancer, treated with bevacizumab, exhibited a significant decrease in T2 signal intensity after initial treatment compared to conventionally treated metastases. Moreover, T2 signal intensity was notably correlated with tumor arterial or late contrast enhancement, as reported by Thüring (29). Correspondingly, pathological examinations demonstrated that angiogenesis inhibitors have the capacity to normalize tumor vasculature, thereby reducing oncotic pressure and normalizing interstitial water content, as noted by Hurwitz (30). In our study, we did not observe a strong correlation between arterial enhancement and T2 hyperintensity. However, it’s important to note that we did not assess late enhancement. Since T2 signal intensity appears to be a crucial factor in therapy monitoring, it may also serve as a predictive marker, warranting further investigation. Nonetheless, it’s crucial to emphasize once more that when evaluating radiological responses to antiangiogenic effects, we should be aware that NELM exhibit different T2 hyperintensity characteristics compared to primary pNET.

Dannecke et al. did not find any evaluated imaging features of hepatic metastases (such as early hyperenhancement, necrosis, or hepatic tumor load) to be prognostic for better progression-free survival in the overall patient cohort, as reported in their study (31). However, when we assessed response-predicting imaging parameters, we found that hepatic tumor burden was significantly lower in responders compared to non-responders (10% versus 30%). Although we did not find imaging features of the NELM themselves, a comparison with the primary tumor might be useful for therapy prediction. NELM in responders displayed a significantly lower ADCmean value compared to the ADC mean value of pNET. In non-responders, T2 hyperintensity was notably higher in NELM compared to pNET. Unlike other studies by Manfredi et al. or Canellas et al., we did not observe significant differences in ductal dilatation between responders and non-responders (8, 32).

In addition the precise analysis of qualitative, and quantitative imaging characteristics of pNET and ts NELM can improve the differentiation regarding other tumors like GIST, and intrapancreatic accessory spleen or chronic mass-forming pancreatitis (33–35), but also may predict treatment response (36, 37).

However, it is essential to acknowledge that the assessment of therapy prediction is limited due to variations in treatments among patients, and this aspect is only a secondary consideration. Additionally, the sample size in our study is relatively small.

Furthermore, there are additional limitations to consider. The primary limitation of this study is its small sample size. Moreover, the retrospective analysis presents a limitation as time intervals between PET/CT scans and MRT scans were heterogeneous, and the subsequent therapies differed among patients. Another limitation is the use of different scanners. While the use of quantitative SUV is well-established for FDG PET/CT using the PERCIST criteria (38), the interpretation of SUV in SSR-PET/CT is more complex, as a reduction in uptake could be attributed to tumor regression or dedifferentiation (39). Therefore, the use of normalized SUV measures by calculating tumor-to-spleen, liver, or blood pool ratios was suggested by various authors (38, 40, 41). Nonetheless, it is important to emphasize that our study primarily aimed to compare NELM and pNET within the same patient. Another limitation of this study is that possible differences between NEC or NET G3 compared to well-differentiated NET were not evaluated.

In conclusion, the study highlights the complexity of pancreatic neuroendocrine tumors and their liver metastases. While conventional ADC and SUV values did not show significant differences, factors such as vascularization, T2 hyperintensity, and tumor burden played crucial roles in distinguishing between NELM and pNET, as well as in predicting treatment responses. These findings emphasize the importance of a multifaceted approach to imaging and treatment planning in patients with these tumors. Tailoring therapy based on these distinctions could lead to more effective and personalized treatment strategies for patients with pancreatic neuroendocrine tumors and liver metastases.

## Data availability statement

The original contributions presented in the study are included in the article/supplementary materials. Further inquiries can be directed to the corresponding author.

## Ethics statement

The studies involving humans were approved by local research ethics committee of the LMU University (approval no. 20-1077). The studies were conducted in accordance with the local legislation and institutional requirements. Written informed consent for participation was not required from the participants or the participants’ legal guardians/next of kin in accordance with the national legislation and institutional requirements. Written informed consent was obtained from the individual(s) for the publication of any potentially identifiable images or data included in this article.

## Author contributions

AH: Writing – original draft, Writing – review & editing. MI: Formal analysis, Writing – original draft, Writing – review & editing. FB: Writing – review & editing. DS: Writing – review & editing. JRü: Writing – review & editing. MZ: Writing – review & editing. VW: Writing – review & editing. JRi: Writing – review & editing. CS-T: Writing – original draft, Writing – review & editing.
